# Defining glioma ecosystem states with AI-driven single-cell and spatial multi-omics: immune-niche reprogramming and therapeutic vulnerabilities

**DOI:** 10.3389/fcell.2026.1943227

**Published:** 2026-10-23

**Authors:** Zhizhen Qin, Yibo Zhang, Bo Wen, Jiaxin Li, Zhenzhen Chen, Xin Li, Dongsheng Kong

**Affiliations:** 1 Senior Department of Neurosurgery, The First Medical Center of PLA General Hospital, Beijing, China; 2 Department of Neurosurgery, The Third Medical Center of Chinese PLA General Hospital, Beijing, China; 3 School of Science and Technology, Hong Kong Metropolitan University, Kowloon, China; 4 Department of Critical Care Medicine, The Third Medical Center of Chinese PLA General Hospital, Beijing, China

**Keywords:** glioblastoma, interpretable machine learning, spatial transcriptomics, therapeutic vulnerability, tumor immune ecosystem

## Abstract

**Objective:**

To define clinically relevant glioma ecosystem states (recurrent spatial configurations of malignant programs and microenvironmental niches) and identify immune-niche-dependent therapeutic vulnerabilities using single-nucleus, spatial, tissue, and machine-learning analyses.

**Methods:**

Public multi-omics datasets were integrated with institutional single-nucleus RNA sequencing from 12 tumors, spatial transcriptomics from 6 IDH-wildtype glioblastomas, multiplex immunofluorescence in 138 FFPE cases, and glioma–macrophage co-culture assays.

**Results:**

Three ecosystem states were identified. The hypoxic myeloid-mesenchymal state showed the highest MES-like, hypoxia, SPP1/APOE myeloid, and temozolomide-resistance scores, the lowest CD8^+^ T-cell density, and the shortest tumor–myeloid distance. In 96 IDH-wildtype glioblastomas, this state independently predicted shorter progression-free survival (HR 2.19, 95% CI 1.22–3.92) and overall survival (HR 2.07, 95% CI 1.12–3.81) after adjustment for major clinical and molecular covariates. Defactinib plus temozolomide reduced viability, colony formation, and migration by 56.0%, 61.3%, and 57.8%, respectively, under macrophage-conditioned culture.

**Conclusion:**

Spatially organized tumor–immune ecosystems provide prognostic information and highlight potentially actionable SPP1–CD44/FAK–SRC signaling in glioblastoma as a candidate target for individualized therapeutic stratification approaches.

## 1 Introduction

Adult diffuse gliomas are among the most biologically heterogeneous and clinically challenging tumors of the central nervous system. Despite advances in surgical techniques, radiotherapy, temozolomide-based chemotherapy, and molecular diagnosis, the prognosis of patients with high-grade glioma, particularly IDH-wildtype glioblastoma, remains poor (Johnson and Lopez-Bertoni, 2024; Tawfeeq et al., 2025). Current integrated classification systems based on IDH mutation, 1p/19q codeletion, MGMT promoter methylation, EGFR amplification, TERT promoter mutation, and other molecular alterations have substantially improved diagnostic accuracy and prognostic stratification. However, these markers do not fully explain the profound intratumoral heterogeneity, immune escape, spatial invasion, treatment resistance, and early recurrence observed in glioblastoma.

Glioma progression is not driven solely by malignant tumor cells. Instead, gliomas develop as complex ecosystems composed of neoplastic cells, resident microglia, infiltrating macrophages, endothelial cells, pericytes, T cells, stromal-like components, and region-specific extracellular matrix structures. Within this ecosystem, malignant cells can occupy multiple transcriptional states, including astrocyte-like, oligodendrocyte progenitor-like, neural progenitor-like, and mesenchymal-like programs. These states are not fixed entities but dynamic and partially overlapping phenotypes that may shift in response to hypoxia, inflammation, therapy-induced stress, and local microenvironmental pressure. Among them, mesenchymal-like transition has been repeatedly associated with hypoxic stress, myeloid infiltration, invasive behavior, and therapeutic resistance (Harwood et al., 2024; Piyadasa et al., 2026; Wu et al., 2023).

The immune microenvironment of glioma is dominated by myeloid cells, including brain-resident microglia and monocyte-derived macrophages. Although these cells are abundant in glioblastoma, they often fail to mount effective antitumor immunity and may instead support tumor growth, extracellular matrix remodeling, angiogenesis, and T-cell exclusion. Traditional M1/M2 polarization is insufficient to describe this diversity. Recent single-cell and spatial studies suggest that distinct myeloid states, such as SPP1- or APOE-associated macrophage-like programs, may form immunosuppressive niches in close proximity to aggressive tumor-cell states. Understanding how these immune populations are spatially organized and functionally connected to malignant cell plasticity is therefore essential for identifying clinically meaningful vulnerabilities (Batchu et al., 2023; Cheng et al., 2025; Mei et al., 2023; Yabo et al., 2024).

Single-cell and single-nucleus RNA sequencing have enabled high-resolution characterization of glioma cellular composition and malignant-state diversity. However, dissociation-based approaches inevitably lose the spatial context required to determine where specific tumor and immune programs occur within the tissue. Spatial transcriptomics addresses this limitation by preserving histological architecture and enabling the localization of transcriptional programs within perinecrotic, hypoxic, perivascular, immune-enriched, and invasive-edge regions. Nevertheless, spatial transcriptomic data require careful integration with single-cell or single-nucleus reference maps, because many spatial platforms capture mixed-cell signals rather than true single-cell resolution (Liu et al., 2024; Onubogu et al., 2024; Sonpatki et al., 2026; Whittle et al., 2025).

Artificial intelligence and interpretable machine-learning methods provide a practical strategy for integrating these multi-layered data. By combining public single-cell and spatial datasets, institutional omics validation, multiplex immunofluorescence, digital pathology, molecular pathology, and clinical outcomes, it is possible to define patient-level ecosystem states that are both biologically interpretable and clinically relevant (Han et al., 2025; Liu et al., 2025; Zheng et al., 2023). Here, we define a glioma “ecosystem state” as a recurrent, spatially organized macro-phenotype driven by specific compositional and functional interactions between malignant cell states and distinct immune-stromal niches. Importantly, such a strategy can reduce experimental cost by using public datasets for discovery, limited institutional sequencing for validation and spatial localization, and FFPE-based tissue cohorts for outcome association. This design is particularly suitable for clinical research teams with access to surgical glioma specimens but limited budgets for large-scale multi-omics or animal experiments.

In this study, we aimed to systematically map these glioma ecosystem states using AI-driven single-nucleus and spatial multi-omics, with a primary focus on immune-niche reprogramming and therapeutic vulnerabilities in IDH-wildtype glioblastoma. We integrated public multi-omics datasets with institutional single-nucleus RNA sequencing, spatial transcriptomics, multiplex immunofluorescence, interpretable machine learning, and glioma cell–macrophage co-culture assays. This workflow was designed to identify malignant and immune cell-state programs, localize high-risk tumor–immune niches, validate ecosystem states in clinical FFPE tissues, and prioritize experimentally testable therapeutic vulnerabilities without the use of animal models.

## 2 Materials and methods

### 2.1 Study design

This animal-free translational study integrated public multi-omics analysis, institutional single-nucleus RNA sequencing, spatial transcriptomics, tissue-based validation, interpretable machine learning, and *in vitro* functional assays (a unified methodological schematic summarizing the data sources, integration steps, and validation workflow is provided in Figure 1). The main focus was IDH-wildtype glioblastoma, while IDH-mutant diffuse gliomas were included only as reference comparators. The workflow utilized public multi-omics datasets for initial discovery, the institutional prospective cohort for single-nucleus cellular-state characterization and spatial niche localization, and the independent retrospective FFPE cohort specifically for clinical and spatial validation of the proposed ecosystem states.

**FIGURE 1:**
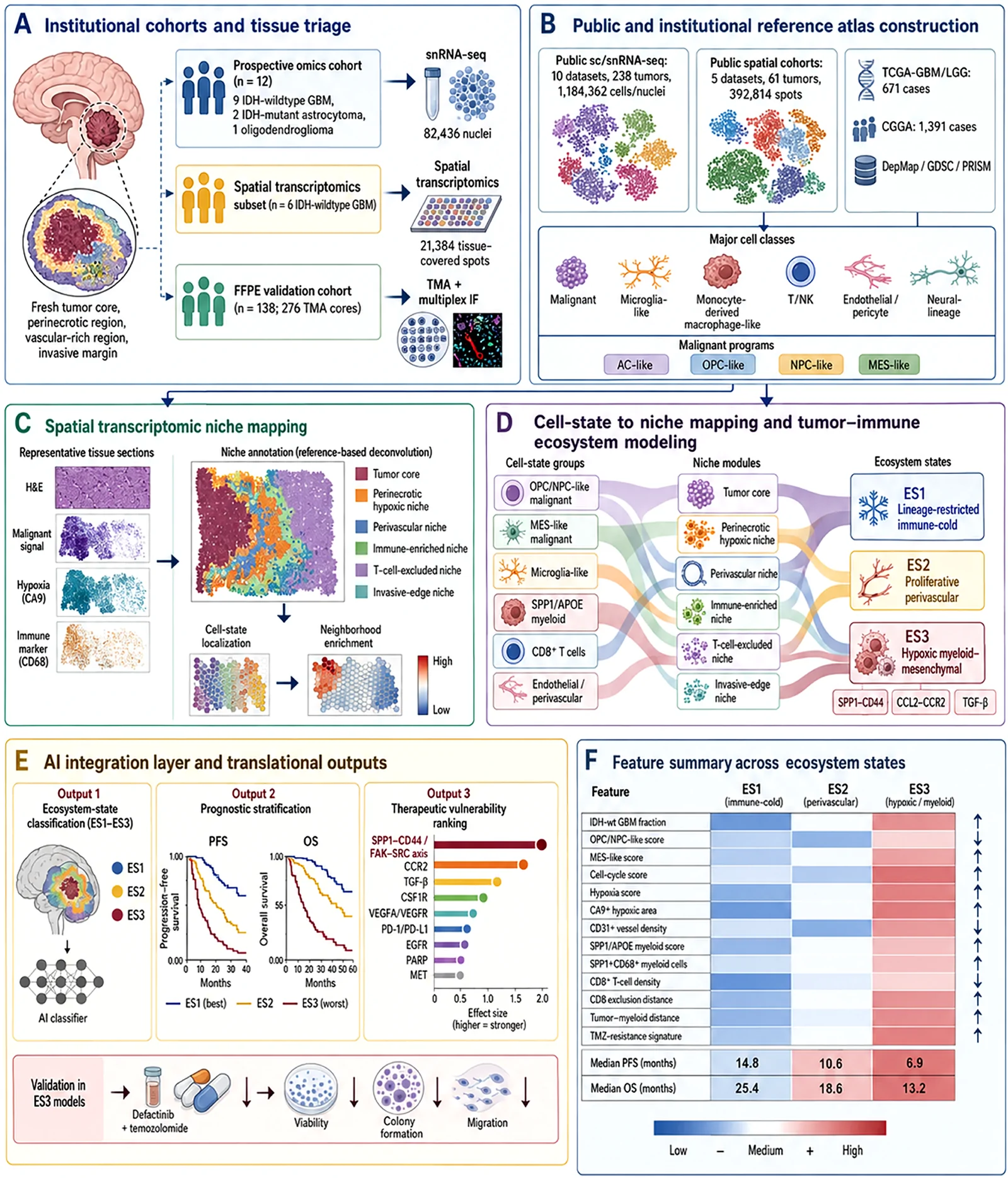
Unified methodological schematic of study architecture, multicohort integration, and the AIdriven workflow for defining glioma ecosystem states. **(A)** Institutional cohorts for singlenucleus RNA sequencing, spatial transcriptomics, and tissue microarrays. **(B)** Reference atlas integrating public and institutional profiles. **(C)** Referencebased spatial deconvolution and anatomical niche mapping. **(D)** Integration of malignant and immune states into ES1–ES3. **(E)** Conceptual workflow linking prognostic stratification, machinelearning classification, therapeutic target screening, and functional validation. Candidate pathway order illustrates the study framework and does not represent final quantitative evidence scores or treatment priority. **(F)** Clinical, molecular, and spatial features across ecosystem states. The schematic was generated using Mermaid.js and assembled for publication.

### 2.2 Patient cohorts and eligibility criteria

The prospective omics cohort included 12 adult patients with newly diagnosed diffuse glioma undergoing surgical resection, including nine IDH‐wildtype glioblastomas, two IDH‐mutant astrocytomas, and one oligodendroglioma. Eligible patients were aged ≥18 years, had no preoperative radiotherapy, chemotherapy, immunotherapy, or targeted therapy, and had sufficient residual tumor tissue after routine pathological diagnosis. The retrospective validation cohort comprised exactly 138 adult diffuse glioma FFPE cases, including 96 IDH-wildtype glioblastomas. While the prospective cohort served to delineate cellular and spatial architectures, this retrospective cohort was dedicated to validating the spatial organization of these states via multiplex immunofluorescence and assessing their prognostic value. Cases with poor tissue quality, incomplete molecular diagnosis, insufficient tumor content, or unavailable survival data were excluded.

### 2.3 Clinical and pathological data collection

Clinical variables included age, sex, preoperative Karnofsky Performance Status score, tumor location, maximal tumor diameter, contrast enhancement, necrosis, extent of resection, postoperative radiotherapy, temozolomide exposure, progression date, death date, and last follow-up date. Pathological and molecular variables included integrated diagnosis, WHO grade, IDH status, ATRX, p53, 1p/19q codeletion, MGMT promoter methylation, TERT promoter mutation, EGFR amplification, and Ki-67 index. Clinically available molecular results were used to reduce additional testing costs.

### 2.4 Public multi-omics data integration

Public glioma single-cell RNA sequencing, single-nucleus RNA sequencing, spatial transcriptomic, bulk transcriptomic, digital pathology, drug-sensitivity, and gene-dependency datasets were collected from open repositories. TCGA-GBM, TCGA-LGG, and CGGA cohorts were used for bulk-level validation, while DepMap, GDSC, and PRISM datasets were used to prioritize druggable vulnerabilities. Public datasets were used for discovery and cross-validation rather than as a substitute for institutional clinical verification (Al-Dalahmah et al., 2023; Fu et al., 2025).

### 2.5 Tumor tissue collection and preservation

Fresh tumor tissue was collected immediately after surgical removal. When feasible, viable tumor core, perinecrotic tissue, vascular-rich region, and invasive margin were separately recorded according to intraoperative navigation and pathological assessment. For single-nucleus sequencing, 20–50 mg of viable tissue was snap-frozen in liquid nitrogen and stored at −80 °C. For spatial transcriptomics, representative tissue was embedded in optimal cutting temperature compound and frozen. Matched tissue was fixed in 10% neutral-buffered formalin for 24–48 h and embedded in paraffin.

### 2.6 Single-nucleus isolation and library preparation

Single-nucleus RNA sequencing was prioritized because glioblastoma tissue is often necrotic and difficult to dissociate into intact viable cells. Frozen tissue was lysed on ice in buffer containing 10 mM Tris-HCl, 10 mM NaCl, 3 mM MgCl_2_, 0.1% NP-40 or IGEPAL CA-630, 1% bovine serum albumin, 1 mM dithiothreitol, and 0.2–0.4 U/μL RNase inhibitor for 5–7 min. Nuclei were filtered through a 40-μm strainer, centrifuged at 500 × g for 5 min at 4 °C, washed twice, and resuspended at 700–1,200 nuclei/μL. Each sample targeted 5,000–8,000 recovered nuclei, with 30,000–50,000 reads per nucleus.

### 2.7 Single-nucleus data processing and quality control

Raw sequencing data were aligned to the human reference genome, and expression matrices were generated using a droplet-based single-nucleus pipeline. Low-quality nuclei with fewer than 300 detected genes, extreme gene or UMI counts, or excessive mitochondrial transcripts were removed. Doublets were detected and removed using Scrublet and DoubletFinder (McGinnis et al., 2019; Wolock et al., 2019), and ambient RNA contamination was corrected when necessary. Data integration and batch-effect correction were performed utilizing Seurat, Harmony, and scVI (Hao et al., 2021; Korsunsky et al., 2019; Lopez et al., 2018). Malignant cells were identified using inferred copy-number alterations, tumor-associated marker expression, and clustering patterns. Differential expression was performed using patient-level pseudobulk analysis.

### 2.8 Cellular-state annotation

Malignant glioma cells were scored for astrocyte-like, oligodendrocyte progenitor-like, neural progenitor-like, mesenchymal-like, cell-cycle, hypoxia, interferon-response, DNA-damage, and therapy-resistance programs. Cell states were analyzed as continuous scores rather than rigid categories. Immune annotation focused on myeloid heterogeneity, including P2RY12/TMEM119-positive microglia-like cells and CD68/APOE/SPP1/FCGR3A-positive monocyte-derived macrophage-like cells. A simplified M1/M2 framework was avoided.

### 2.9 Spatial transcriptomics

Six IDH-wildtype glioblastoma specimens with adequate viable tumor, perinecrotic tissue, vascular proliferation, and invasive regions were selected for spatial transcriptomics. Fresh-frozen tissue sections were cut at 10 μm thickness and mounted on spatial capture slides. One section from each case was used for spatial transcriptomic profiling, while adjacent sections were used for hematoxylin–eosin staining and multiplex immunofluorescence. Libraries were prepared according to the platform workflow and sequenced to approximately 50,000–100,000 reads per tissue-covered spot. Low-quality, non-tissue, and artifact-heavy spots were excluded.

### 2.10 Spatial deconvolution and niche identification

Spatial transcriptomic data were integrated with institutional and public single-nucleus reference maps. Cell-type and cell-state composition within spatial spots was quantitatively estimated using cell2location, RCTD, and Tangram (Biancalani et al., 2021; Cable et al., 2022; Kleshchevnikov et al., 2022). Candidate niches were defined by histology and transcriptomic features, including tumor core, perinecrotic hypoxic niche, perivascular niche, immune-enriched niche, T-cell-excluded niche, and invasive-edge niche. Co-localization between mesenchymal-like malignant cells and SPP1/APOE-associated myeloid cells was quantified using neighborhood enrichment, spot-level correlation, and distance-based analysis (Sankowski et al., 2024; Sattiraju et al., 2023).

### 2.11 Tumor–immune communication and vulnerability prioritization

Tumor–immune communication and ligand–receptor interactions were inferred using CellChat and NicheNet (Browaeys et al., 2020; Jin et al., 2021). Ligand–receptor pairs were retained only when they showed plausible sender–receiver expression, spatial proximity, downstream target-gene support, and reproducibility across datasets. Therapeutic vulnerabilities were prioritized by integrating enrichment in high-risk cell states, localization within adverse niches, survival association, glioma cell-line dependency, drug-sensitivity evidence, and inhibitor availability. Candidate pathways included SPP1–CD44, CCL2–CCR2, TGF-β signaling, integrin–extracellular matrix signaling, and hypoxia-related signaling.

### 2.12 Tissue microarray and multiplex immunofluorescence

Tissue microarrays used pathologistselected 1.5–2.0mm cores from viable tumor centers and invasive/peripheral FFPE regions. Figure 4D used six representative independent patients per ecosystem state (18 total), selected for preserved architecture, adequate analyzable tissue/staining, and absence of major folds, tissue loss, necrotic artifacts, or severe autofluorescence. Panel 1 comprised DAPI, SOX2, OLIG2, CD44, CA9, and CD31; panel 2 comprised DAPI, IBA1, CD68, P2RY12, SPP1, and CD8. Antigen retrieval used citrate (pH 6.0) or TrisEDTA (pH 9.0) for 15–20 min. Primary antibodies (1:100–1:500) were incubated overnight at 4 °C, followed by tyramide amplification and DAPI counterstaining (0.5–1 μg/mL).

### 2.13 Digital pathology and spatial quantification

Whole-slide fluorescent images were acquired using a multispectral or fluorescence scanner and analyzed with QuPath or equivalent software. Image preprocessing included tissue segmentation, autofluorescence correction, channel unmixing, and artifact exclusion. Quantitative features included marker-positive cell density, marker intensity, double-positive cell proportion, myeloid-cell density, CD8 T-cell density, vessel density, CA9-positive hypoxic area, nearest-neighbor distance between tumor and myeloid cells, CD8 T-cell exclusion distance, cellular composition within 50 μm of CD31-positive vessels, and myeloid-cell enrichment within 100 μm of CA9-positive regions.

### 2.14 *In vitro* glioma cell–macrophage co‐culture

LN229 and T98G or U251 glioma cells were cultured in Dulbecco’s modified Eagle’s medium with 10% fetal bovine serum and 1% penicillin–streptomycin. THP-1 monocytes were cultured in RPMI-1640 with the same supplements and differentiated using 20 ng/mL phorbol 12-myristate 13-acetate for 24 h, followed by 24 h recovery in PMA-free medium. If macrophage induction was insufficient, PMA was optimized within 20–50 ng/ mL. Macrophage-conditioned glioma models were established using macrophage-conditioned medium or a 0.4-μm Transwell non- contact co-culture system. Treatment groups included control, temozolomide, candidate inhibitor, combination treatment, macrophage-conditioned control, macrophage-conditioned temozolomide, macrophage-conditioned inhibitor, and macrophage-conditioned combination treatment. Temozolomide was tested at 50–500 μM, and the candidate inhibitor was tested at 0.1–10 μM or according to reported IC50 values.

### 2.15 Functional assays and statistical analysis

Cell viability was measured by CCK-8 assays at 24, 48, and 72 h. Colony formation was assessed by seeding 500–1,000 cells per well in six-well plates for 10–14 days, followed by fixation with 4% paraformaldehyde and staining with 0.1% crystal violet. Migration was evaluated using 8-μm Transwell chambers. RT-qPCR measured CD44, CHI3L1, SERPINE1, OLIG2, PDGFRA, SOX2, SPP1, CCL2, TGFB1, and selected downstream genes. Western blotting used RIPA-extracted protein supplemented with protease and phosphatase inhibitors, with 20–40 μg protein loaded per lane. Membranes were probed with primary antibodies against phosphorylated FAK (p-FAK), total FAK, phosphorylated SRC (p-SRC), total SRC, and GAPDH (as an internal loading control), followed by incubation with appropriate HRP-conjugated secondary antibodies and chemiluminescent detection. Each experiment included at least three independent biological replicates. Continuous and categorical variables were analyzed using appropriate parametric or non-parametric tests. Survival was evaluated using Kaplan–Meier analysis, log-rank tests, and Cox regression. For ecosystem-state modeling and prognostic stratification, an extreme gradient boosting (XGBoost) classifier was developed to integrate the multidimensional spatial and transcriptomic features. Hyperparameter optimization was conducted via grid search coupled with 10-fold cross-validation to prevent overfitting. The resulting machine-learning models were robustly assessed by bootstrap resampling, C-index, time-dependent AUC, and calibration curves. Furthermore, feature importance and directional effects were decoded using SHapley Additive exPlanations (SHAP) to ensure biological interpretability. Custom analytical scripts, algorithmic configurations, and associated data are available from the corresponding authors upon reasonable request. Multiple testing was corrected using the Benjamini–Hochberg method, and twosided P < 0.05 was considered significant.

## 3 Results

### 3.1 Cohort construction and multi-omics workflow

Figure 1A summarizes specimen allocation across the institutional cohorts, delineating their distinct functional roles. The prospective omics cohort, utilized for high-resolution cellular and spatial mapping, included 12 treatment-naïve diffuse gliomas (nine IDH-wildtype glioblastomas, two IDH-mutant astrocytomas, and one oligodendroglioma). Single-nucleus sequencing generated 82,436 high-quality nuclei. Six IDH-wildtype glioblastomas underwent spatial transcriptomic profiling, yielding 21,384 tissue-covered spots, whereas 138 FFPE cases contributed 276 tissue-microarray cores for multiplex immunofluorescence and clinical validation. Tissue sampling incorporated viable tumor, perinecrotic, vascular-rich, and invasive-margin regions.

Figure 1B shows integration of the institutional data with public single-cell, spatial, bulk-transcriptomic, gene-dependency, and drug-sensitivity resources. The resulting reference atlas resolved six major cellular compartments and four malignant programs, including AC-like, OPC-like, NPC-like, and MES-like states. Figure 1C outlines reference-based spatial deconvolution and the identification of tumor-core, perinecrotic hypoxic, perivascular, immune-enriched, T-cell-excluded, and invasive-edge niches. Figure 1D links malignant and immune programs to these anatomical niches and shows their convergence into three ecosystem states. ES3 was preferentially connected to MES-like malignant cells, SPP1/APOE-associated myeloid cells, hypoxic niches, and SPP1–CD44, CCL2–CCR2, and TGF-β signaling. Figure 1E presents the principal analytical outputs: ecosystem-state classification, prognostic stratification, therapeutic-vulnerability ranking, and functional validation. Figure 1F summarizes the progressive increase in mesenchymal, hypoxic, myeloid, immune-exclusion, and temozolomide-resistance features from ES1 to ES3.

Table 1 details the clinical and molecular characteristics. Median age was 56.5 years in the prospective cohort, 60.5 years in the spatial subset, and 58.0 years in the FFPE cohort. IDH-wildtype glioblastoma accounted for 75.0%, 100.0%, and 69.6% of the respective cohorts. Gross-total resection was achieved in 56.5% of the FFPE cohort, 84.1% received postoperative radiotherapy, and 81.2% received temozolomide. Median follow-up was 24.6 months, with median progression-free survival and overall survival of 12.1 (95% CI, 10.3–14.6) and 22.7 (95% CI, 19.4–27.8) months, respectively.

**TABLE 1:** Baseline clinical and molecular characteristics of the institutional glioma cohorts.

Characteristics	Prospective snRNA-seq cohort (n = 12)	Spatial transcriptomics subset (n = 6)	FFPE validation cohort (n = 138)
Age, years	56.5 (45.0–64.8)	60.5 (53.3–66.5)	58.0 (49.0–67.0)
Male sex	7 (58.3)	4 (66.7)	79 (57.2)
Preoperative KPS score	80 (70–90)	80 (70–85)	80 (70–90)
Maximal tumor diameter, cm	4.8 ± 1.2	5.1 ± 1.1	4.6 ± 1.4
Contrast-enhancing tumor	10 (83.3)	6 (100.0)	109 (79.0)
Radiological necrosis	8 (66.7)	6 (100.0)	87 (63.0)
Frontal lobe involvement	4 (33.3)	2 (33.3)	46 (33.3)
Temporal lobe involvement	3 (25.0)	2 (33.3)	37 (26.8)
Parietal/occipital involvement	3 (25.0)	1 (16.7)	32 (23.2)
Deep or multifocal lesion	2 (16.7)	1 (16.7)	23 (16.7)
IDH-wildtype glioblastoma	9 (75.0)	6 (100.0)	96 (69.6)
IDH-mutant astrocytoma	2 (16.7)	0 (0.0)	26 (18.8)
Oligodendroglioma, IDH-mutant and 1p/19q-codeleted	1 (8.3)	0 (0.0)	16 (11.6)
MGMT promoter methylation	5 (41.7)	2 (33.3)	59 (42.8)
TERT promoter mutation	8 (66.7)	5 (83.3)	83 (60.1)
EGFR amplification	6 (50.0)	4 (66.7)	72 (52.2)
ATRX loss	3 (25.0)	0 (0.0)	34 (24.6)
p53 overexpression	4 (33.3)	1 (16.7)	49 (35.5)
Ki-67 index, %	30 (18–42)	38 (28–46)	26 (14–38)
Gross total resection	7 (58.3)	3 (50.0)	78 (56.5)
Subtotal resection or biopsy	5 (41.7)	3 (50.0)	60 (43.5)
Postoperative radiotherapy	10 (83.3)	6 (100.0)	116 (84.1)
Temozolomide treatment	10 (83.3)	6 (100.0)	112 (81.2)
Median follow-up, months	14.8 (9.6–20.5)	13.2 (8.8–18.4)	24.6 (16.2–39.5)
Median PFS, months	​	​	12.1 (95% CI, 10.3–14.6)
Median OS, months	​	​	22.7 (95% CI, 19.4–27.8)

Table 2 summarizes the analytical scale. Ten public single-cell or single-nucleus datasets contributed 1,184,362 cells or nuclei, and five public spatial cohorts contributed 392,814 tissue-covered spots. Bulk validation included 671 TCGA and 1,391 CGGA cases. Institutional profiling generated 82,436 nuclei from 12 tumors and 21,384 spatial spots from six IDH-wildtype glioblastomas.

**TABLE 2:** Public and institutional multi-omics datasets included in the study.

Data source	Dataset type	Number of datasets	Samples/cases	Cells, nuclei, spots, or images after QC	Main analytical purpose
Public glioma scRNA-seq/snRNA-seq cohorts	Single-cell/single-nucleus transcriptomics	10	238 tumors	1,184,362 cells/nuclei	Reference atlas construction; malignant and immune cell-state discovery
Public glioma spatial transcriptomic cohorts	Spatial transcriptomics	5	61 tumors	132 tissue sections; 392,814 tissue-covered spots	Spatial niche reference; tumor–immune co-localization validation
TCGA-GBM and TCGA-LGG	Bulk RNA-seq and clinical outcomes	2	671 cases	671 transcriptomic profiles	Bulk-level validation of ecosystem signatures
CGGA glioma cohorts	Bulk transcriptomics and survival data	2	1,391 cases	1,391 transcriptomic profiles	External survival validation and subtype comparison
Public digital pathology datasets	H&E whole-slide images	3	842 cases	842 evaluable slides	Histology-associated ecosystem feature extraction
DepMap	Gene dependency	1	68 glioma-lineage cell lines	Genome-wide dependency scores	Druggable vulnerability prioritization
GDSC and PRISM	Drug-sensitivity datasets	2	74 glioma-lineage cell lines	Drug-response profiles	Candidate inhibitor screening and ranking
Institutional prospective cohort	snRNA-seq	1	12 patients	82,436 nuclei	Institutional validation of cellular programs
Institutional spatial subset	Spatial transcriptomics	1	6 IDH-wildtype GBM cases	21,384 tissue-covered spots	Localization of GBM immune niches
Institutional FFPE cohort	Tissue microarray and mIF	1	138 patients	276 tissue cores	Clinical and spatial validation of ecosystem states

### 3.2 Single-nucleus transcriptomic profiling defines the cellular architecture of adult diffuse gliomas

Figure 2A shows the integrated UMAP of 82,436 nuclei, resolving malignant cells, microglia-like cells, monocyte-derived macrophage-like cells, T/NK cells, endothelial cells/pericytes, and non-malignant neural-lineage cells. Figure 2B confirms that these compartments were detected across molecular subtypes, although the monocyte-derived macrophage-like compartment was visibly expanded in IDH-wildtype glioblastoma.

**FIGURE 2:**
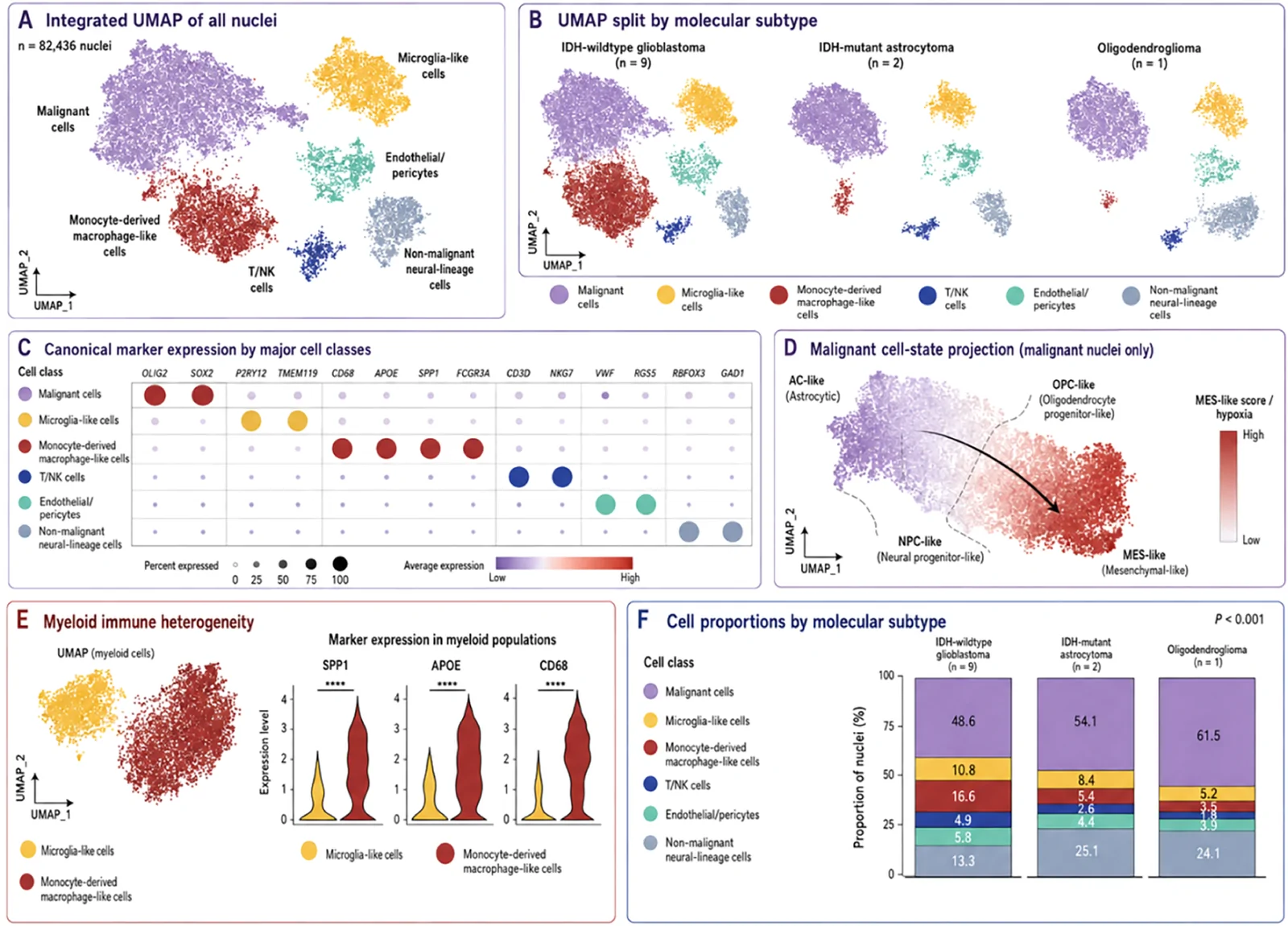
Single-nucleus atlas resolves malignant cell-state plasticity and myeloid immune heterogeneity across adult diffuse gliomas. **(A,B)** Integrated UMAP embeddings of 82,436 nuclei colored by major cell classes **(A)** and stratified by molecular subtype **(B)**. **(C)** Dot plot illustrating canonical marker expression used for cell-class annotation; dot size indicates the percentage of expressing cells, and color intensity represents average scaled expression. **(D)** Trajectory projection of malignant nuclei highlighting the continuum toward the MES-like state, overlaid with scaled hypoxia scores. **(E)** UMAP sub-clustering of myeloid populations and corresponding violin plots of marker distribution (****adjusted P < 0.0001). **(F)** Stacked bar plots comparing cell-class proportions across subtypes. High-contrast, colorblind-friendly palettes were utilized in all UMAP embeddings to enhance visual discrimination and accessibility.

Figure 2C validates cell-class annotation. Malignant nuclei expressed OLIG2 and SOX2; microglia-like cells expressed P2RY12 and TMEM119; monocyte-derived macrophage-like cells expressed CD68, APOE, SPP1, and FCGR3A; T/NK cells expressed CD3D and NKG7; endothelial cells/pericytes expressed VWF and RGS5; and neural-lineage cells expressed RBFOX3 and GAD1. Figure 2D demonstrates a continuous malignant-state distribution connecting AC-like, OPC-like, NPC-like, and MES-like programs. Increasing MES-like and hypoxia scores occurred along the same transcriptional continuum, supporting state plasticity rather than mutually exclusive malignant subtypes.

Figure 2E separates P2RY12/TMEM119-positive microglia-like cells from CD68/APOE/SPP1-positive monocyte-derived macrophage-like cells. SPP1, APOE, and CD68 expression was markedly higher in the latter population. Figure 2F shows significant variation in cellular composition across molecular subtypes (adjusted P < 0.001, Benjamini–Hochberg method). Monocyte-derived macrophage-like cells represented 16.6% of nuclei in IDH-wildtype glioblastoma, compared with 5.4% in IDH-mutant astrocytoma and 3.5% in oligodendroglioma.

Table 3 confirms sequencing quality and cellular composition. The median yield was 6,873 nuclei per sample, with 2,268 detected genes and 4,956 UMIs per nucleus. The estimated doublet-removal rate was 5.5%, and the median mitochondrial transcript proportion was 2.8%. Malignant cells represented 48.6% of nuclei in IDH-wildtype glioblastoma, 54.1% in IDH-mutant astrocytoma, and 61.5% in oligodendroglioma. Total myeloid cells represented 27.4%, 13.8%, and 8.7%, respectively, supporting preferential myeloid accumulation in IDH-wildtype glioblastoma.

**TABLE 3:** Single-nucleus RNA sequencing quality metrics and cell-type composition.

Metrics	IDH-wildtype GBM (n = 9)	IDH-mutant astrocytoma (n = 2)	Oligodendroglioma (n = 1)	Total cohort (n = 12)
Nuclei retained after QC	63,218	13,184	6,034	82,436
Median nuclei per sample	6,912 (6,108–7,642)	6,592 (6,214–6,970)	6,034	6,873 (6,096–7,516)
Median genes per nucleus	2,214 (1,826–2,648)	2,362 (1,942–2,781)	2,514 (2,011–2,866)	2,268 (1,847–2,701)
Median UMIs per nucleus	4,812 (3,625–6,390)	5,106 (3,914–6,842)	5,438 (4,125–7,028)	4,956 (3,701–6,566)
Estimated doublets removed, %	5.8 (4.9–6.7)	5.1 (4.5–5.6)	4.8	5.5 (4.8–6.4)
Median mitochondrial transcript proportion, %	2.9 (2.1–4.2)	2.5 (1.8–3.7)	2.2	2.8 (2.0–4.0)
Malignant glioma cells, %	48.6	54.1	61.5	50.7
Microglia-like cells, %	10.8	8.4	5.2	9.8
Monocyte-derived macrophage-like cells, %	16.6	5.4	3.5	13.9
Total myeloid cells, %	27.4	13.8	8.7	23.7
T/NK cells, %	4.9	2.6	1.8	4.2
Endothelial cells/pericytes, %	5.8	4.4	3.9	5.4
Non-malignant neural-lineage cells, %	13.3	25.1	24.1	16

### 3.3 Malignant cell-state plasticity and myeloid immune reprogramming define glioma ecosystem states

Three reproducible ecosystem states were identified in the FFPE cohort. As shown in Table 4, ES1 was designated the lineage-restricted immune-cold state and included 48 tumors. It was characterized by a high OPC/NPC-like score, low MES-like and hypoxia scores, low SPP1/APOE myeloid activity, and the highest CD8-positive T-cell density. ES1 also showed the longest tumor–myeloid nearest-neighbor distance and the lowest activity of the SPP1–CD44, CCL2–CCR2, and TGF-β pathways.

**TABLE 4:** Molecular, immune, and spatial features defining glioma ecosystem states.

Features	ES1: Lineage-restricted immune-cold state (n = 48)	ES2: Proliferative perivascular state (n = 42)	ES3: Hypoxic myeloid-mesenchymal state (n = 48)	P value
IDH-wildtype GBM cases, n (%)	18 (37.5)	30 (71.4)	48 (100.0)	<0.001
MES-like malignant score, z-score	−0.62 ± 0.41	0.18 ± 0.46	0.91 ± 0.52	<0.001
OPC/NPC-like malignant score, z-score	0.74 ± 0.48	0.22 ± 0.44	−0.38 ± 0.47	<0.001
Cell-cycle score, z-score	−0.28 ± 0.39	0.82 ± 0.51	0.36 ± 0.46	<0.001
Hypoxia score, z-score	−0.41 ± 0.36	0.34 ± 0.43	1.08 ± 0.57	<0.001
CA9-positive hypoxic area, %	6.8 (3.9–10.6)	15.2 (9.8–22.4)	31.5 (23.7–42.8)	<0.001
CD31-positive vessel density, vessels/mm^2^	18.4 (12.5–25.6)	34.7 (26.2–45.3)	27.9 (20.1–38.5)	<0.001
Perivascular niche score, z-score	−0.21 ± 0.37	0.88 ± 0.49	0.39 ± 0.44	<0.001
SPP1/APOE myeloid score, z-score	−0.58 ± 0.40	0.21 ± 0.45	1.12 ± 0.54	<0.001
SPP1+CD68^+^ myeloid cells, cells/mm^2^	42 (25–68)	86 (53–126)	172 (118–241)	<0.001
CD8^+^ T-cell density, cells/mm^2^	112 (72–156)	86 (51–132)	49 (28–83)	<0.001
CD8 T-cell exclusion distance, μm	38 (24–55)	56 (37–79)	92 (64–128)	<0.001
Tumor–myeloid nearest-neighbor distance, μm	74 (52–103)	48 (32–71)	25 (16–39)	<0.001
SPP1–CD44 interaction score	0.28 ± 0.16	0.57 ± 0.21	0.86 ± 0.24	<0.001
CCL2–CCR2 interaction score	0.24 ± 0.15	0.49 ± 0.18	0.79 ± 0.22	<0.001
TGF-β pathway activity score	0.31 ± 0.18	0.54 ± 0.22	0.83 ± 0.25	<0.001
Temozolomide-resistance signature, z-score	−0.31 ± 0.38	0.22 ± 0.41	0.72 ± 0.49	<0.001

ES2, the proliferative perivascular state, included 42 tumors and showed the highest cell-cycle score, CD31-positive vessel density, and perivascular-niche score. Its hypoxic, mesenchymal, and myeloid features were intermediate between ES1 and ES3.

ES3, the hypoxic myeloid-mesenchymal state, included 48 tumors, all of which were IDH-wildtype glioblastomas. ES3 showed the highest MES-like score, hypoxia score, CA9-positive area, SPP1/APOE myeloid score, and temozolomide-resistance signature. It also had the highest density of SPP1-positive/CD68-positive myeloid cells, the shortest tumor–myeloid distance, the lowest CD8-positive T-cell density, and the greatest CD8 T-cell exclusion distance. SPP1–CD44, CCL2–CCR2, and TGF-β interaction scores were all maximal in ES3, supporting a coordinated hypoxic, myeloid-rich, and mesenchymal program.

### 3.4 Spatial transcriptomics reveals hypoxic, perivascular, perinecrotic, and immune-enriched niches in IDH-wildtype glioblastoma

Figure 3A identifies six spatially organized compartments across the IDH-wildtype glioblastoma sections. Tumor-core regions were bordered by perinecrotic hypoxic areas, while perivascular, immune-enriched, T-cell-excluded, and invasive-edge niches occupied distinct but partially overlapping tissue regions.

**FIGURE 3:**
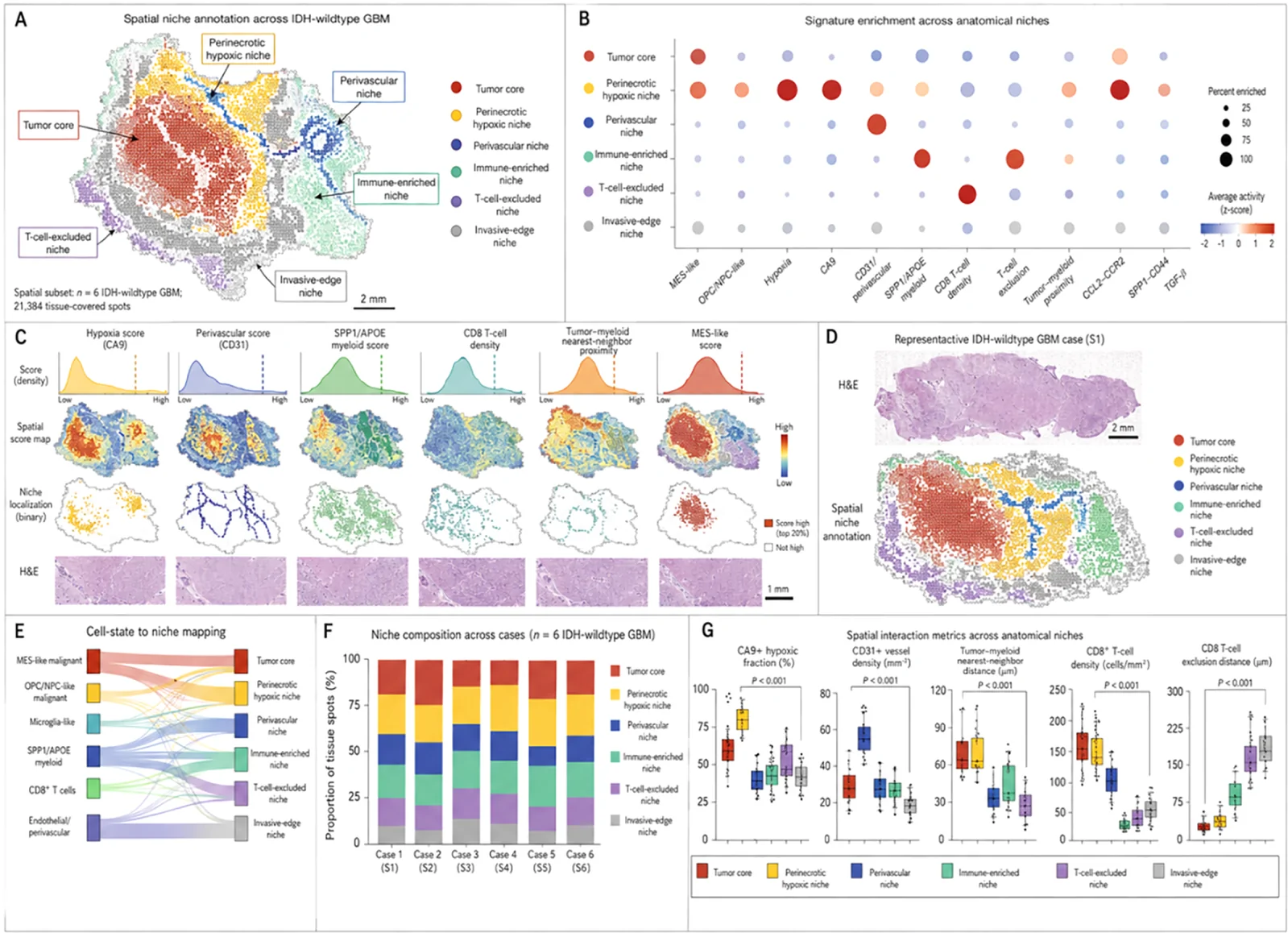
Spatial transcriptomics delineates anatomical niches and tumor–immune neighborhood interactions in IDH-wildtype glioblastoma. **(A)** Representative spatial map annotating six distinct anatomical niches. **(B)** Dot plot showing transcriptomic signature enrichment across defined niches (dot size represents percent enriched; color intensity indicates average z-score). **(C)** Spatial feature plots aligning hypoxia, perivascular, myeloid, and T-cell signals with corresponding H&E morphology. **(D)** Alignment of spatial niche annotation with H&E histology in a representative sample. **(E–G)** Quantification of cell-state mapping, inter-case niche composition, and spatial neighborhood interaction metrics. Scale bars, 1 mm or 2 mm as indicated. Color palettes in spatial maps were selected using colorblind-safe configurations to ensure visual accessibility.

Figure 3B shows niche-specific enrichment of molecular and immune signatures. Perinecrotic niches were enriched for hypoxia, CA9, MES-like activity, and SPP1–CD44 signaling. Perivascular niches showed maximal CD31-associated activity, whereas immune-enriched niches displayed strong SPP1/APOE myeloid activity and tumor–myeloid proximity. T-cell-excluded regions had the strongest exclusion signal and limited CD8-positive T-cell enrichment.

Figure 3C spatially localizes the major scores. Hypoxia and MES-like signals co-accumulated around perinecrotic regions, CD31 activity followed vascular structures, and SPP1/APOE-associated myeloid activity was concentrated within hypoxic and immune-enriched areas. CD8-positive T cells were spatially depleted from regions with high hypoxia and MES-like activity. Figure 3D confirms these patterns in a representative tumor section by aligning H&E morphology with the spatial niche annotation.

Figure 3E maps cell states to anatomical compartments. MES-like malignant cells and SPP1/APOE-associated myeloid cells preferentially mapped to perinecrotic hypoxic and immune-enriched niches, whereas endothelial/perivascular cells mapped predominantly to the perivascular compartment. Figure 3F shows intertumoral variation in niche proportions across the six spatially profiled tumors, while preserving the same major niche classes in every case.

Figure 3G quantifies spatial differences. CA9-positive area was highest in perinecrotic hypoxic regions, and CD31-positive vessel density was highest in perivascular regions. Tumor–myeloid nearest-neighbor distance was shortest in immune-enriched regions. CD8-positive T-cell density was lowest and exclusion distance was greatest in T-cell-excluded regions. All principal niche comparisons remained significant at an adjusted P < 0.001 following multiple-testing correction.

### 3.5 Multiplex immunofluorescence validates ecosystem states in the institutional FFPE cohort

Figure 4A shows representative whole-section multiplex immunofluorescence images across the three ecosystem states. ES1 displayed limited CD44, CA9, SPP1, and CD68 staining, with relatively preserved CD8-positive T-cell signals. ES2 contained prominent CD31-positive vascular structures and intermediate hypoxic and myeloid signals. ES3 showed extensive CD44-positive tumor regions, increased CA9 expression, and dense SPP1-positive/CD68-positive myeloid infiltration.

**FIGURE 4:**
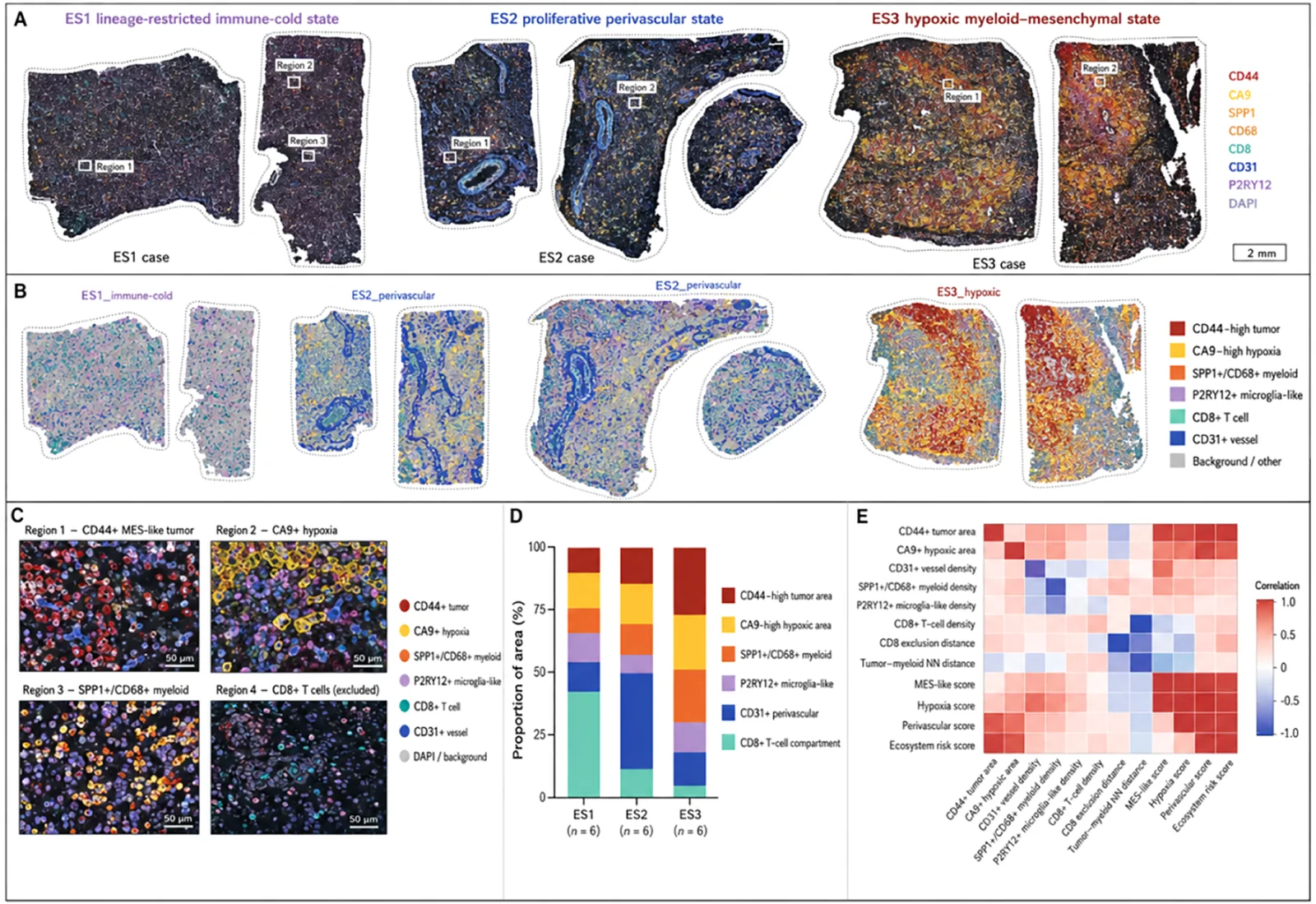
Multiplex immunofluorescence validates spatial organization of hypoxic myeloid–mesenchymal ecosystem states in the institutional FFPE cohort. **(A,B)** Representative multispectral tissue images **(A)** and markerdefined spatial maps **(B)** across ES1–ES3; scale bars, 2 mm. **(C)** Enlarged views of functional niches; scale bars, 50 μm. **(D)** Markerdefined tissue proportions in six representative FFPE specimens from independent patients per state(18 patients total), not fields of view or technical replicates. Specimens had preserved tumor/edge architecture, sufficient analyzable tissue, and adequate staining, without major folds, tissue loss, necrotic artifacts, or severe autofluorescence; the same imageanalysis workflow was used across groups. **(E)** Correlations of spatial features with ecosystem risk. Channel pseudocolors were optimized for contrast and separation of overlapping markers.

Figure 4B converts the whole-section images into marker-defined spatial maps. ES1 was dominated by immune-cold and microglia-like regions, ES2 showed extensive perivascular organization, and ES3 contained large, spatially overlapping CD44-high, CA9-high, and SPP1-positive/CD68-positive compartments.

Figure 4C provides high-magnification validation. CD44-positive MES-like tumor cells were abundant in aggressive tumor regions, CA9-positive cells localized to hypoxic areas, and SPP1-positive/CD68-positive myeloid cells were positioned close to CD44-positive tumor cells. CD8-positive T cells were sparse within these ES3 regions and accumulated predominantly outside the CD44-high and CA9-high compartments.

Figure 4D summarizes marker-defined tissue composition. The relative contributions of CD44-high tumor, CA9-high hypoxia, and SPP1-positive/CD68-positive myeloid compartments increased from ES1 to ES3. In contrast, the CD8-positive T-cell compartment decreased across the same sequence. The CD31-positive perivascular compartment was most prominent in ES2.

Figure 4E shows coordinated associations among tissue features. CD44-positive area, CA9-positive area, SPP1-positive/CD68-positive myeloid density, MES-like score, hypoxia score, and ecosystem risk score were positively correlated. CD8-positive T-cell density was inversely related to these high-risk features, whereas CD8 exclusion distance was positively associated with them. Tumor–myeloid distance was inversely associated with MES-like, hypoxic, and myeloid characteristics.

Quantitatively, median SPP1-positive/CD68-positive myeloid density increased from 42 cells/mm^2^ in ES1 to 86 cells/mm^2^ in ES2 and 172 cells/mm^2^ in ES3. Median CD8-positive T-cell density decreased from 112 to 86 and 49 cells/mm^2^, respectively. CA9-positive area increased from 6.8% to 15.2% and 31.5%. Tumor–myeloid distance decreased from 74 to 48 and 25 μm, whereas CD8 exclusion distance increased from 38 to 56 and 92 μm.

### 3.6 Ecosystem states are associated with clinical outcomes and improve prognostic stratification

Table 5 quantifies the prognostic associations in the 96-patient IDH-wildtype glioblastoma subgroup. In univariable analysis, ES3 was associated with a 2.83-fold higher progression risk than ES1. After adjustment for age, KPS, extent of resection, MGMT promoter methylation, and completion of standard chemoradiotherapy, ES3 remained independently associated with progression-free survival (HR, 2.19; 95% CI, 1.22–3.92; P = 0.008). ES2 was not significantly different from ES1 after adjustment.

**TABLE 5:** Associations between ecosystem states and clinical outcomes.

Variables	Median PFS, months	Univariable HR for PFS (95% CI)	P value	Multivariable HR for PFS (95% CI)	P value	Median OS, months	Multivariable HR for OS (95% CI)	P value
ES1: Lineage-restricted immune-cold state (n = 18)	14.8	Reference	​	Reference	​	25.4	Reference	​
ES2: Proliferative perivascular state (n = 30)	10.6	1.52 (0.83–2.78)	0.172	1.34 (0.71–2.54)	0.365	18.6	1.29 (0.66–2.52)	0.452
ES3: Hypoxic myeloid-mesenchymal state (n = 48)	6.9	2.83 (1.63–4.92)	<0.001	2.19 (1.22–3.92)	0.008	13.2	2.07 (1.12–3.81)	0.02
Age ≥60 years	8.4	1.61 (1.07–2.43)	0.022	1.38 (0.89–2.15)	0.151	15.1	1.44 (0.90–2.29)	0.126
KPS score <80	7.6	1.89 (1.22–2.93)	0.004	1.56 (0.97–2.51)	0.066	13.8	1.68 (1.02–2.77)	0.042
Subtotal resection or biopsy	7.8	1.74 (1.15–2.64)	0.009	1.49 (0.94–2.37)	0.091	14.6	1.53 (0.94–2.50)	0.087
MGMT unmethylated	7.2	1.92 (1.25–2.96)	0.003	1.73 (1.09–2.74)	0.02	13.9	1.81 (1.11–2.95)	0.017
Standard chemoradiotherapy completed	11.9	0.58 (0.36–0.93)	0.024	0.64 (0.39–1.04)	0.073	20.8	0.61 (0.36–1.03)	0.064
Continuous ecosystem risk score	​	1.78 (1.36–2.34)	<0.001	1.52 (1.14–2.04)	0.004	​	1.47 (1.08–2.01)	0.015

MGMT-unmethylated status independently predicted shorter progression-free survival (HR, 1.73; 95% CI, 1.09–2.74; P = 0.020). The continuous ecosystem risk score remained significant after multivariable adjustment (HR per unit increase, 1.52; 95% CI, 1.14–2.04; P = 0.004). For overall survival, ES3 was associated with an adjusted HR of 2.07 compared with ES1 (P = 0.020). KPS below 80 and MGMT-unmethylated status were also associated with shorter overall survival. To quantify this added prognostic value, we compared the predictive performance of the machine-learning-derived ecosystem model against a standard clinical baseline (incorporating age, KPS, extent of resection, and MGMT status). The integrated model, which combined the continuous ecosystem risk score with clinical variables, yielded a significantly higher concordance index (C-index) of 0.74 (95% CI, 0.69–0.79) compared to the clinical baseline alone (C-index, 0.65; 95% CI, 0.60–0.70; P = 0.014). Furthermore, time-dependent ROC analysis for 12-month overall survival demonstrated a notable improvement, with the AUC increasing from 0.67 in the baseline model to 0.78 in the integrated model. These metrics definitively indicate that ecosystem-state classification provides robust prognostic information beyond conventional clinical and molecular variables.

### 3.7 AI-based therapeutic vulnerability prioritization and *in vitro* functional validation

Figure 5A presents the multivariable Cox models in the 96-patient IDH-wildtype glioblastoma subgroup. For progression-free survival, ES3 remained independently associated with progression compared with ES1 (HR, 2.19; 95% CI, 1.22–3.92; P = 0.008). MGMT-unmethylated status (HR, 1.73; 95% CI, 1.09–2.74; P = 0.020) and the continuous ecosystem risk score (HR per unit, 1.52; 95% CI, 1.14–2.04; P = 0.004) were also independently associated with progression.

**FIGURE 5:**
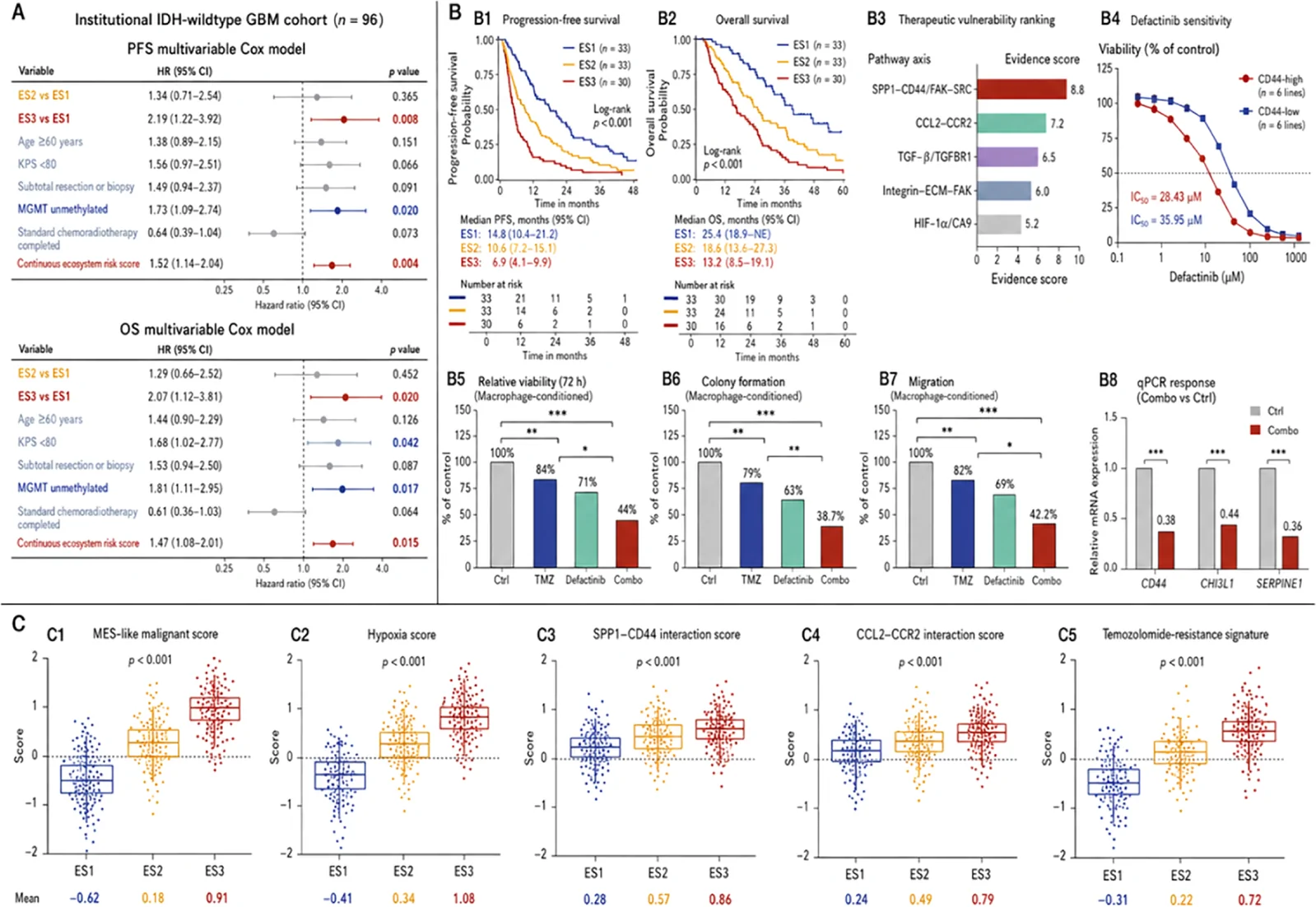
Prognostic associations and therapeutic vulnerabilities of the hypoxic myeloid–mesenchymal ecosystem state. **(A)** Multivariable Cox models for PFS and OS in IDH‐wildtype glioblastoma. **(B)** PFS/OS curves with risk tables (B1,B2), pathway evidence scores (B3), and defactinib dose responses (B4). B4 shows mean ± SD across six independent cell lines per group at 0–75 μM; lines connect means, and IC50 values are retained as reported. B5–1B8 show viability, colony formation, migration, and qPCR expression under macrophageconditioned treatments, with SD from biological triplicates. Significance: *P < 0.05, **P < 0.01, ***P < 0.001. **(C)** Individual observations and box plots of multidimensional signatures across ES1–ES3 (C1–C5); boxes show the IQR and center lines the median. Colors distinguish groups/treatments.

For overall survival, ES3 remained an independent adverse factor (HR, 2.07; 95% CI, 1.12–3.81; P = 0.020). KPS below 80 (HR, 1.68; 95% CI, 1.02–2.77; P = 0.042), MGMT-unmethylated status (HR, 1.81; 95% CI, 1.11–2.95; P = 0.017), and the ecosystem risk score (HR per unit, 1.47; 95% CI, 1.08–2.01; P = 0.015) were independently associated with shorter survival.

Figure 5B1 shows progressive separation of the progression-free survival curves from ES1 to ES3. Median progression-free survival was 14.8 months (95% CI, 10.4–21.2) in ES1, 10.6 months (95% CI, 7.2–15.1) in ES2, and 6.9 months (95% CI, 4.1–9.9) in ES3 (log-rank P < 0.001). Figure 5B2 shows the same ordered pattern for overall survival, with median values of 25.4 (95% CI, 18.9–not estimable [NE]), 18.6 (95% CI, 13.6–27.3), and 13.2 (95% CI, 8.5–19.1) months, respectively (log-rank P < 0.001).

Figure 5B3 ranks therapeutic vulnerabilities by integrated evidence. The SPP1–CD44/FAK–SRC axis received the highest score at 8.8, followed by CCL2–CCR2 at 7.2, TGF-β/TGFBR1 at 6.5, integrin–ECM–FAK at 6.0, and HIF-1α/CA9 at 5.2. To functionally validate the dependence of mesenchymal-like glioma cells on the SPP1–CD44 signaling cascade, we targeted its primary intracellular mechanotransducer, FAK, using defactinib. Figure 5B4 shows greater defactinib sensitivity in CD44-high glioma lines, with an estimated IC_50_ of 28.43 μM compared with 35.95 μM in CD44-low lines.

Under macrophage-conditioned conditions, Figure 5B5 shows that relative viability at 72 h was 100% in controls, 84% with temozolomide, 71% with defactinib, and 44% with combination treatment. Figure 5B6 shows corresponding colony-formation values of 100%, 79%, 63%, and 38.7%. Figure 5B7 shows that migration decreased from 100% in controls to 82% with temozolomide, 69% with defactinib, and 42.2% with the combination. Thus, combined treatment reduced viability, colony formation, and migration by 56.0%, 61.3%, and 57.8%, respectively.

Figure 5B8 shows that combination treatment reduced CD44, CHI3L1, and SERPINE1 mRNA expression to 0.38-, 0.44-, and 0.36-fold of control levels, respectively. Furthermore, Western blot analysis confirmed that the combined treatment markedly attenuated the protein expression levels of phosphorylated FAK and phosphorylated SRC relative to their total protein counterparts (Supplementary Figure S1). Figures 5C1–C5 confirm the biological specificity of the high-risk state. ES3 had the highest MES-like score (0.91), hypoxia score (1.08), SPP1–CD44 score (0.86), CCL2–CCR2 score (0.79), and temozolomide-resistance score (0.72), whereas ES1 consistently had the lowest values (all adjusted P < 0.001).

Table 6 summarizes the final vulnerability ranking. The SPP1–CD44/FAK–SRC axis had the strongest multi-layer support and the most consistent *in vitro* response. CCL2–CCR2, TGF-β/TGFBR1, integrin–ECM–FAK, and HIF-1α/CA9 signaling showed moderate or supportive evidence, whereas the PDGFRA/OPC-like program was restricted to selected PDGFRA-high models and was classified as exploratory.

**TABLE 6:** Prioritized therapeutic vulnerabilities and experimental validation results.

Rank	Candidate vulnerability	Dominant ecosystem state	Main cellular source	Spatial niche	Representative inhibitor	DepMap/GDSC/PRISM support	*In vitro* validation summary	Overall evidence
1	SPP1–CD44/FAK–SRC axis	ES3: Hypoxic myeloid-mesenchymal state	SPP1+CD68^+^ myeloid cells and CD44^+^ MES-like tumor cells	Perinecrotic and immune-enriched niches	Defactinib or FAK inhibitor	Dependency enriched in mesenchymal-like glioma lines; higher predicted sensitivity in CD44-high models	Combination with TMZ reduced viability by 56.0%, colony formation by 61.3%, and migration by 57.8%; CD44, CHI3L1, and SERPINE1 were downregulated	Strong
2	CCL2–CCR2 signaling	ES3: Hypoxic myeloid-mesenchymal state	Tumor cells and monocyte-derived macrophage-like cells	Myeloid-enriched niche	CCR2 inhibitor	Associated with macrophage-rich transcriptional profiles and poor survival signatures	Inhibition reduced macrophage-conditioned migration by 34.5% and decreased CCL2-related inflammatory markers	Moderate
3	TGF-β/TGFBR1 signaling	ES3 and ES2	Myeloid cells, endothelial cells, and MES-like tumor cells	Perivascular and hypoxic niches	SB431542 or TGFBR1 inhibitor	Linked to ECM remodeling and mesenchymal-transition signatures	Inhibition reduced SERPINE1 and TGFB1 expression and partially restored TMZ sensitivity	Moderate
4	Integrin–ECM–FAK signaling	ES2: Proliferative perivascular state	Perivascular tumor cells, endothelial cells, and stromal-like cells	Perivascular niche	FAK/integrin pathway inhibitor	Dependency associated with adhesion and invasion programs	Reduced transwell migration by 41.2% and suppressed focal-adhesion-related proteins	Moderate
5	HIF-1α/CA9 hypoxia axis	ES3: Hypoxic myeloid-mesenchymal state	Hypoxic malignant cells	Perinecrotic hypoxic niche	PX-478 or hypoxia-pathway inhibitor	Enriched in hypoxia-high, treatment-resistant transcriptional profiles	Reduced CA9 and VEGFA expression; modest effect on viability alone but enhanced TMZ response	Supportive
6	PDGFRA/OPC-like lineage program	ES1: Lineage-restricted immune-cold state	OPC-like malignant cells	Tumor-core lineage niche	PDGFR inhibitor	Dependency limited to PDGFRA-high models	Growth inhibition observed only in PDGFRA-high glioma cells; weak effect in macrophage-conditioned models	Exploratory

## 4 Discussion

This study defined three biologically distinct glioma ecosystem states by integrating single-nucleus transcriptomics, spatial transcriptomics, tissue-based validation, clinical outcomes, and interpretable machine learning. The principal finding was that the hypoxic myeloid-mesenchymal state represented the most aggressive ecosystem. It was characterized by strong MES-like tumor programs, extensive CA9-positive hypoxia, dense SPP1-positive and CD68-positive myeloid infiltration, reduced CD8-positive T-cell density, and increased exclusion of cytotoxic T cells from tumor regions. These features were accompanied by the shortest tumor–myeloid distance, the strongest SPP1–CD44, CCL2–CCR2, and TGF-β signaling activities, and the highest temozolomide-resistance signature (Ji et al., 2025). By contrast, the lineage-restricted immune-cold state retained stronger OPC-like and NPC-like programs, lower hypoxic and myeloid activity, and more favorable clinical outcomes. The proliferative perivascular state showed an intermediate phenotype dominated by cell-cycle activity and vascular organization.

The single-nucleus analysis confirmed that adult diffuse gliomas are not composed of stable, mutually exclusive cellular phenotypes. Malignant cells occupied a continuous transcriptional spectrum connecting AC-like, OPC-like, NPC-like, and MES-like states. This pattern supports a model in which local environmental pressure promotes state transition rather than simply selecting fixed clones. The enrichment of MES-like programs in hypoxic and myeloid-rich regions generates the hypothesis that malignant plasticity might be modulated by microenvironmental signals, though our static multi-omics approach cannot definitively establish direct causality. The greater proportion of monocyte-derived macrophage-like cells in IDH-wildtype glioblastoma also indicates that recruitment of peripheral myeloid populations is a defining feature of aggressive disease.

Spatial analysis added an essential anatomical dimension to these transcriptional findings. Hypoxia was concentrated around perinecrotic regions, vascular activity was localized to perivascular compartments, and SPP1/APOE-associated myeloid programs were enriched in immune-dense and hypoxic areas (Pai et al., 2025). The close spatial proximity between malignant cells and myeloid cells in the high-risk ecosystem strongly implicates potential active reciprocal signaling rather than passive coexistence (Zhao et al., 2025). However, we recognize that this remains a working hypothesis pending direct mechanistic validation in complex dynamic models. In parallel, the increased distance between CD8-positive T cells and tumor cells offers a potential spatial correlate for immune exclusion, although the dynamic processes of T-cell trafficking and functional exhaustion cannot be fully captured by these cross-sectional tissue snapshots. These observations indicate that the biological effect of immune infiltration depends on cellular identity and spatial organization, not simply on total immune-cell abundance.

The tissue validation cohort demonstrated that these ecosystem states were clinically relevant. The hypoxic myeloid-mesenchymal state was associated with substantially shorter progression-free and overall survival. Its association with progression remained significant after adjustment for age, performance status, extent of resection, MGMT promoter methylation, and standard chemoradiotherapy. The continuous ecosystem risk score also retained independent prognostic value. While current integrated diagnostic frameworks excel at defining initial oncogenic drivers (e.g., IDH, MGMT, and TERT status), they rely on static, tumor-centric genomic profiles that often fail to capture the dynamic microenvironmental pressures driving adaptive resistance. Our findings suggest that spatially informed ecosystem assessment can complement and, in predicting therapeutic failure, potentially surpass conventional molecular classifications. By evaluating the multicellular network—specifically the convergence of hypoxia, mesenchymal tumor plasticity, and SPP1-positive macrophage infiltration—this model captures treatment-relevant spatial interactions not represented by tumor genotype alone, providing a critical translational bridge toward individualized microenvironmental interventions.

Multi-evidence prioritization highlighted the SPP1–CD44/FAK–SRC axis as a top candidate therapeutic vulnerability warranting further *in vivo* investigation. MES-like glioma cell lines showed greater FAK dependency, and CD44-high lines were more sensitive to defactinib. In macrophage-conditioned cultures, combining defactinib with temozolomide produced the strongest reductions in viability, colony formation, and migration. The combination also suppressed CD44, CHI3L1, SERPINE1, phosphorylated FAK, and phosphorylated SRC. These results support a model in which macrophage-derived SPP1 reinforces mesenchymal and invasive tumor behavior through CD44-dependent FAK–SRC signaling, while FAK inhibition partially restores temozolomide sensitivity (Lu et al., 2026). We acknowledge that while pharmacological FAK inhibition effectively disrupts this mechanotransduction cascade, future studies utilizing targeted genetic ablation (e.g., CRISPR/Cas9) or neutralizing antibodies specifically directed against SPP1 and CD44 are required to orthogonally validate the upstream receptor-ligand dependency.

An additional strength is the staged design, in which public datasets supported discovery, institutional specimens confirmed cellular and spatial patterns, and functional assays tested the convergent target. This structure improved coherence while limiting sequencing and experimental costs. However, several methodological limitations must be acknowledged. First, the spatial transcriptomics platform used captures multi-cellular spots rather than true single-cell resolution; thus, our niche composition mapping relies on computational deconvolution frameworks, which inherently introduce algorithmic estimation biases. Second, regarding the integrated machine-learning strategies, while our AI classifier utilized robust cross-validation to mitigate overfitting, such data-driven models remain intrinsically sensitive to the distribution of their training cohorts. Rigorous external validation across diverse clinical demographics and independent multi-omics platforms is necessary to establish the broader generalizability of the ecosystem risk score. Finally, although SHAP analysis enhances algorithmic transparency, these computational models fundamentally capture multidimensional correlations rather than definitive causality, further emphasizing the necessity for subsequent mechanistic validation.

## 5 Conclusion

This study established an integrated framework for defining clinically relevant glioma ecosystem states by combining public multi-omics resources, institutional single-nucleus sequencing, spatial transcriptomics, multiplex immunofluorescence, interpretable machine learning, and focused functional validation. Three ecosystem states were identified, among which the hypoxic myeloid-mesenchymal state showed the strongest mesenchymal, hypoxic, myeloid, immune-exclusion, and temozolomide-resistance features and independently predicted unfavorable progression-free and overall survival in IDH-wildtype glioblastoma. Multi-layer evidence further implicated the SPP1–CD44/FAK–SRC axis as a potentially actionable interaction between macrophage-like cells and malignant glioma cells. Inhibition of FAK with defactinib enhanced temozolomide activity and suppressed glioma-cell viability, clonogenic growth, migration, and mesenchymal signaling under macrophage-conditioned conditions. These findings suggest that spatially organized tumor–immune ecosystems provide information beyond conventional molecular classification and could ultimately inform biologically informed prognostic assessment and individualized therapeutic stratification in glioblastoma.

## Data Availability

The data supporting the findings of this study are available from the corresponding authors upon reasonable request.
