# Supplementary material for: Defining glioma ecosystem states with AI-driven single-cell and spatial multi-omics: immune-niche reprogramming and therapeutic vulnerabilities
Source: Front Cell Dev Biol. 2026 Oct 23;14:1943227. doi: 10.3389/fcell.2026.1943227 (PMC13739698; doi:10.3389/fcell.2026.1943227)
Supplement: Supplementary file 1 [file Supplementaryfile1.docx]

**Supplementary Figure S1**

Pharmacological inhibition of FAK suppresses the SPP1-CD44/FAK-SRC signaling cascade in macrophage-conditioned glioma cells.
